# 40 Years of Pfs48/45 Research as a Transmission-Blocking Vaccine Target of *Plasmodium falciparum* Malaria

**DOI:** 10.4269/ajtmh.21-1320

**Published:** 2022-07-11

**Authors:** Robert W. Sauerwein, Jordan Plieskatt, Michael Theisen

**Affiliations:** ^1^ TropIQ Health Sciences, Nijmegen, The Netherlands;; ^2^ Department for Congenital Disorders, Statens Serum Institut, Copenhagen, Denmark;; ^3^ Centre for Medical Parasitology, Department of Immunology and Microbiology, University of Copenhagen, Copenhagen, Denmark

## Abstract

In the early 1980s, Richard Carter was among the first researchers to identify the sexual stage-specific Pfs48/45 protein, leading to the identification of target epitopes. Carter predicted its tertiary conformation while involved in a number of studies on naturally acquired sexual stage-specific antibodies. Pfs48/45 is a cysteine-rich surface protein of sexual stages of *Plasmodium falciparum* that plays a critical role in male gamete fertility. Antibodies against Pfs48/45 prevent parasite development in the mosquito vector, and therefore prevent the spread of malaria in the population. Since the gene was sequenced in the early 1990s, Pfs48/45 has been considered a prime target candidate for a malaria transmission-blocking vaccine. However, major manufacturing challenges—in particular, difficulty realizing satisfactory yields of a properly folded protein for the induction of functional antibodies—delayed clinical development significantly. These challenges were met roughly 20 years later. The first clinical trial with a Pfs48/45 subunit vaccine (R0.6C) was started in the Netherlands in early 2021. The excellent contributions to the long and winding path of Pfs48/45 research by Richard Carter are well recognized and are an integrated part of his seminal contributions to unraveling *Plasmodium* sexual stage biology.

## IDENTIFICATION AND CHARACTERIZATION OF Pfs48/45

The first evidence for the existence of sexual stage specific *Plasmodium* proteins came from studies conducted by Richard Carter and colleagues in the beginning of the 1980s. Immunizations of rodents with sexual stages of *Plasmodium falciparum* induced antibodies with transmission-blocking activity, precipitating targeting gamete proteins earlier identified in avian malaria, *Plasmodium gallinacium*.^1^^,^^2^ Subsequently, the Meuwissen group from the Netherlands further characterized the molecular weights using a panel of gamete-specific monoclonal antibodies (mAbs), including those from Carter, showing the presence of a doublet (48- and 45-kDa molecular weight under non-reducing conditions) among other target proteins.^3^^,^^4^ All mAbs recognized both the 45- and 48-kDa component of the doublet, hence the terminology of Pfs48/45. The significance of this doublet became very clear, because specific antibodies were relatively potent in blocking oocyst formation by preventing fertilization in the mosquito midgut. By then, it was also shown that proper protein conformation was critical, depending upon intact disulfide bonds, as reducing sodium dodecyl sulfate–treatment abolished the binding and blocking activity completely by anti-Pfs48/45 mAbs.^3^ The gene—cloned, sequenced, and expressed by Kocken et al.^5^—encodes for a hydrophobic, non-repetitive protein of 448 amino acid residues.

Pfs48/45 is synthesized exclusively during gametocytogenesis from days 2 to 3 onward, continuing through until gametes emerge from erythrocytes in the mosquito midgut. The protein is membrane bound via a glycophosphatidylinositol anchor and linked to Pfs230.^6^ Because Pfs48/45 is not expressed on the membrane of circulating red blood cells infected with gametocytes, specific antibodies remain non-functional until rapid transition takes place into gametes once inside the mosquito midgut, thereby blocking sporogony. Already in the early studies, it was clear that Pfs48/45 was involved in the fertilization process, because specific antibodies prevented the development of oocysts in the mosquito midgut.^3^ Although expressed on the surface membrane of both male and females gametes, biological function appeared to be a critical component in determining male fertility. Male gametes of Pfs48/45 gene-deletion mutants were unable to adhere and penetrate female gametes, whereas disruption in female gametes did not affect their fertility.^7^

**Figure 1.**
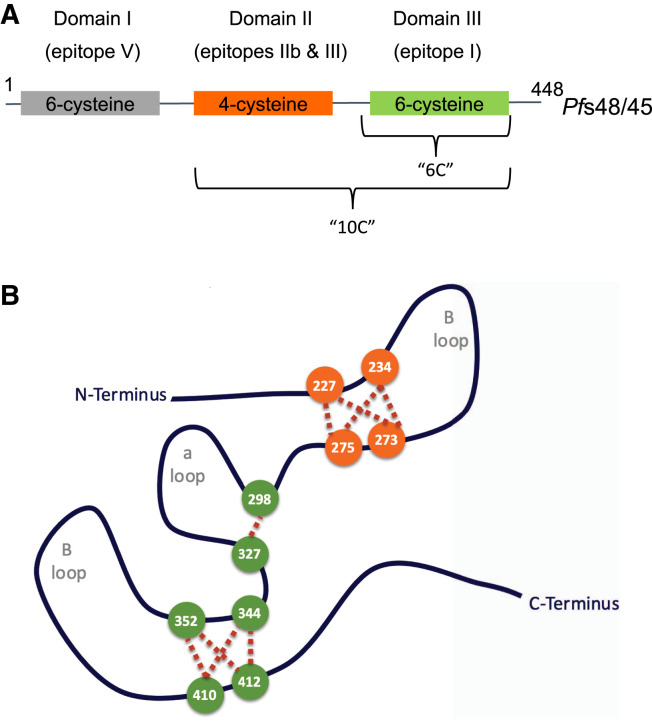
(**A**) Schematic representation of *Pfs*45/48. Domains containing six or four cysteines are shown as boxes. Domain II and domain III together encompass the “10C” fragment as explored, and domain III is denoted by “6C.” 6C (green box) is the current focus of *Pfs*48/45 domains in vaccine candidates R0.6C and ProC6C. (**B**) Schematic representation of the middle and C-terminal domains of *Pfs*48/45 as modified from Carter et al.,^8^ and indicating disulfide pairing as investigated and reported previously.^10^ The proposed disulfide bond for domains II (orange circles) and III (green circles) was deduced from the analysis of non-reduced and reduced trypsin-digested R0.10C. Amino acid numbers are denoted according to amino acid residue no. 1 of *Pfs*48/45.^10^ Disulfide connectivity is indicated by the dashed red lines. The ambiguities are the result of the close proximity of some of the cysteine pairs. The exact and expected pairing of cysteines for Pfs48/45 was resolved by structural analysis of the 6-Cys domain, where Cys1-Cys2 (Cys298-Cys327, as noted here), Cys3-Cys6 (Cys344-Cys412, as noted here), and Cys4-Cys5 (Cys352-410, as noted here) form the cysteine pairs.^16^

As for its structure, Pfs48/45 is a member of the six-cysteine *Plasmodium* family characterized by a unique pattern of a conserved cysteine structure with 6 of 10 members in sexual stages. Pfs48/45 has three domains containing six, four, and six cysteine residues, respectively (Figure 1A^8–10^). Carter and colleagues postulated a specific arrangement of these domains, with repetitive motifs and internal and outfacing loops (Figure 1B).^8^ A single (6-Cys) s48/45 domain adopts a β-sandwich fold, where two disulfide-bonds are formed between each β-sheet and one disulfide bond is outside this core structure.^11^ Using mass spectrometry, we confirmed that the hypothetical structure predicted by Carter et al, including disulfide bond connectivity and tertiary structure, was indeed correct (Figure 1B).^8^^,^^9^ This disulfide bond arrangement provides an explanation for the conformation-dependent exposure of epitopes for transmission-blocking anti-Pfs48/45 mAbs.^12^

Further insight into the structural organization of Pfs48/45 was obtained by using a panel of independent anti-Pfs48/45 mAbs, including ones generated by Carter et al. by protein digestion, expression of truncated forms, and use of cysteine mutations and refolding assays.^13^^,^^14^ At least four different epitopes were found that block or reduce malaria transmission.^15^ The C-terminal module was recognized by mAbs against epitope I as well as a middle and N-terminal module. The cysteines in the central and C-terminal modules appear to be crucial for proper presentation of the transmission-blocking epitopes.

The C‐terminal domain containing six cysteines and epitope I is still the target of the most potent transmission-blocking mAb: 85RF45.1.^16^ A fully humanized version of mAb 85RF45.1(TB31F) has been manufactured for clinical development and is currently being tested in a clinical phase I study (https://clinicaltrials.gov/ct2/show/NCT04238689) for safety and tolerability. More recently, the crystalized structure of this Pfs48/45 C-terminal 6-Cys domain has been determined, allowing for detailed information for structure-based vaccine development.^10^^,^^17^

## NATURALLY ACQUIRED ANTI-Pfs48/45 ANTIBODIES

The first evidence for naturally acquired antibodies to block transmission was reported by Graves, Carter, and coworkers^18^ in donors living in Papua New Guinea, and included anti-Pfs48/45 antibodies. Many studies followed over the years that examined the potential predictive value of anti-Pfs48/45 antibodies for transmission-blocking activity of field sera, as reviewed by Muthui et al.^19^ Sexual stage-specific antibodies targeting specific epitopes are acquired rapidly in gametocyte carriers, but wane rapidly after infection.^18^

The contribution of anti-Pfs48/45 antibodies to blocking activity was often suggestive, with positive correlations in a number of different malaria-endemic regions,^4^^,^^18^^,^^20^^–^^27^ but not always present.^19^^,^^28^ Clearly other targets may also come into play—in particular, Pfs230—and their relative functional contribution may depend on the induced antibody profile as a function of gametocyte prevalence, in addition to a number of other determining factors.^29^ Only recently, circumstantial evidence for transmission-reducing activity of naturally acquired anti-Pfs48/45 was shown by affinity purification of specific antibodies from endemic sera.^27^ In any case, the potential for boosting of vaccine-induced immune responses during natural infection would enhance vaccine efficacy in the field, but this obviously requires further study.

One important aspect is that the sequence of Pfs48/45 from laboratory and wild-type parasite lines is relatively conserved.^30^^,^^31^ Sequence diversity is generally low across strains of *P. falciparum*, and much lower than observed for some asexual-stage target antigens.^32^ This is obviously relatively good news for antibody-based transmission-blocking intervention strategies. Because non-synonymous mutations, however, do occur, heterologous protection should be ensured and remain a key subject of clinical studies. Illustratively, the potent anti-Pfs48/45 mAb 45.1 is able to block oocyst development of many natural gametocyte carriers involving diverse parasite strains and multiclonal infections, whereas an anti-Pfs230 mAb shows a restricted blocking capacity.^33^

## DEVELOPMENT OF TRANSMISSION-BLOCKING VACCINES BASED ON Pfs48/45

Previously, clinical development of Pfs48/45-based transmission blocking vaccines has been stalled because of insufficient yields of recombinant protein that folds into the native structure required for induction of transmission-blocking antibodies (reviewed Theisen et al.^34^). The complex nature of Pfs48/45, which includes three domains with multiple cysteines, further complicates recombinant protein production. Proper folding of cysteine-rich proteins depends on correct disulfide bond formation (Figure 1).^35^ As such, several attempts to produce full-length or sub-unit domains in eukaryotic expression systems have led to low yields of properly folded protein.^34^ The limited capability of eliciting transmission-blocking antibodies may relate to shielding of epitopes by post-translational modifications (i.e., glycosylation or inability of the heterologous expression system to recreate the native disulfide bonds properly). Only recently, production of full-length Pfs48/45 was successful in *Drosophila Schneider*-2 cells, and was recognized by conformation-dependent antibodies.^17^

## CLINICAL DEVELOPMENT OF Pfs48/45 R0.6C

To avoid possible interference of post-translational modifications, we used *Lactococcus lactis* for expression and focused on the production of properly folded Pfs48/45 domains (Table1). Initial development explored the expression of the central four-cysteine and distal six-cysteine domains together (10C) (Figure 1A), with the carrier protein (R0) derived from the *P. falciparum* glutamate-rich protein (reviewed by Theisen et al.^36^) as R0.10C.^35^ However, yields could be increased further by truncation of the 10C fragment into the six-cysteine-containing C-terminal domain (6C) alone (Figure 1A).^37^^,^^38^ The 6C fragment recognized by conformation depended on mAb 45.1 and was fused with the R0 protein to facilitate expression and proper folding further to become R0.6C. Although various recombinant subdomains of the native Pfs48/45 have been shown to elicit functional antibodies in multiple animal species, R0.6C was selected as a prime candidate for downstream clinical development.^15^^,^^35^ Immunogenicity and potency of R0.6C was tested in combination with a large number of adjuvant formulations in small rodents. Adsorption to Alhydrogel^®^ (Croda International, Denmark) increased immunogenicity, whereas the addition of Matrix-M^TM^ (Novavax AB, Uppsala, Sweden) further enhanced induction of high concentrations of functional transmission-blocking antibodies.^39^ These studies led to the planned drug product configuration: R0.6C adsorbed to Alhydrogel and with the inclusion of the saponin-based adjuvant Matrix-M, to be mixed at bedside prior to administration. Furthermore, supported by excellent pre-clinical safety data, R0.6C/Alhydrogel and R0.6C/Alhydrogel + Matrix-M adjuvant candidate vaccines entered first-in-human clinical trials in The Netherlands in 2021 (NCT04862416). In addition, the R0.6C vaccine candidate will be entering the phase of clinical testing in West Africa as an integral part of the Malaria Transmission Blocking Consortium (pftbv.org) funded by the European and Developing Countries Clinical Trial Partnership.

**Table 1:** Current status of clinical testing of Pfs48/45-based vaccine candidates

Variable	*Pfs*48/45 candidate	
	R0.6C	ProC6C
Application	TBV: Sporogonic stage	TBV and AIV: Sporogonic and sporozoite stages
*Pfs*48/45 component	C-terminal 6-cysteine domain	C-terminal 6-cysteine domain
Additional proteins	GLURP “R0”	*Pfs*230: “Pro” domain CSPc: repeat region
Drug product composition	R0.6C/AlOH R0.6C/AlOH + Matrix-M	ProC6C/AlOH ProC6C/AlOH + Matrix-M
Development summary	R0.6C was designed by SSI (Denmark) and Radboudum* (the Netherlands†), and manufactured under cGMP at SSI (Denmark).	The chimeric antigen was developed by fusing the TBV leads *Pfs*230 and *Pfs*48/45 with a CSP linker sequence.* ProC6C was manufactured under cGMP at SSI (Denmark).
Clinical evaluation	Trial 1: R0.6C/AlOH/Matrix-M at Radboudum* (the Netherlands) (NCT04862416) Trial 2: GRAS, Burkina Faso, planned in 2022 Trial 3: USTTB, Mali planned in 2022	Trial 1: ProC6C/AlOH/Matrix-M by GRAS, Burkina Faso, planned in 2022 Trial 2: USTTB, Mali planned in 2022
Clinical doses	30 μg R0.6C ± 15 μg Matrix-M 100 μg R0.6C ± 50 μg Matrix-M	30 μg ProC6C ± 15 μg Matrix-M 100 μg ProC6C ± 50 μg Matrix-M

AIV = anti-infection vaccine; cGMP = current good manufacturing practice; TBV = transmission-blocking vaccine.

* Pf circumsporozoite protein: NANPNVDPNANPNVDPNANPNVDPNANPNANPNANP.

† With financial support of PATH (https://www.path.org) under Grant OPP1108403 from the Bill & Melinda Gates Foundation and in part by Grant NNF14CC0001 and the European Union’s Horizon 2020 Research and Innovation Program under grant agreement no. 733273.

## ProC6C AS A POTENTIAL CHIMERIC MULTISTAGE VACCINE CANDIDATE

As a potential next-generation candidate, ProC6C has been produced in *L. lactis* by combining the 6C fragment of Pfs48/45 with the transmission-blocking domains of Pfs230, thereby replacing the R0 domain with Pro domain of Pf230 (Table 1).^40^^,^^41^ The Pfs230 “Pro” domain was linked to the pertinent 6C domain of Pfs48/45 by a short protein linker sequence that includes the *P. falciparum* circum-sporozoite protein major and minor repeat units as expressed in sporozoites.

In preclinical models, this candidate elicited high titers of transmission-blocking antibodies as well as antibodies that block hepatocyte invasion by sporozoites comparable to those elicited by full-length circum-sporozoite proteins.^42^ Thus, ProC6C as a chimeric “multistage” malaria vaccine is potentially capable of protecting against a malaria infection while also blocking transmission of the parasite in the population. The ProC6C vaccine candidate has been produced under current good manufacturing practice using a process built upon the success of expression and purification of R0.6C. Similar to that of R0.6C, ProC6C/Alhydrogel ± Matrix-M adjuvant will be entering the phase of clinical testing in West Africa as integral part of the Malaria Transmission Blocking Consortium (pftbv.org) funded by the European and Developing Countries Clinical Trial Partnership.

## CONCLUSION

The biology and epidemiology of malaria transmission has shown to be an intriguing and challenging subject over many decades. Richard Carter was one of the pioneers with a strong voice to emphasize its paramount importance for effective malaria control and, eventually, elimination. Because circulating gametocytes are clinically silent and, as such, are not involved in clinical signs and symptoms, studies on malaria transmission received relatively little attention and priority until 2007. In that year, Bill and Melinda Gates expressed the ambition to eradicate malaria.^43^ Since that moment, malaria transmission and the clinical development of a transmission-blocking vaccine has been an integral part of the malaria research agenda. Now, 15 years later, the first transmission-blocking vaccine based on the Pfs48/45 protein, R0.6C, as well as transmission-blocking mAb 32F1 have entered the phase of clinical testing. Furthermore, a clinical trial with the multistage vaccine candidate ProC6C has been planned in Africa. As such, the ambitions and memorable contributions of Richard Carter are to become translated into clinical tools that eventually may realize effective interruption of malaria transmission and spread of the disease.
